# Cerebrospinal fluid of chronic osteoarthritic patients induced interleukin-6 release in human glial cell-line T98G

**DOI:** 10.1186/s12871-020-00985-0

**Published:** 2020-03-25

**Authors:** Weiling Liu, Chunmei Li, Francis Chee Kuan Tan, Hong Jye Neo, Yiong Huak Chan, Chian-Ming Low, Tat Leang Lee

**Affiliations:** 1 Department of Anaesthesia, National University Hospital, National University Health System, 5 Lower Kent Ridge Road, Singapore, 119074 Singapore; 2 Department of Anaesthesia, Yong Loo Lin School of Medicine, National University of Singapore, 5 Lower Kent Ridge Road, Singapore, 119074 Singapore; 3 Biostatistics Unit, Yong Loo Lin School of Medicine, National University of Singapore, Singapore, Singapore; 4 Department of Pharmacology, Yong Loo Lin School of Medicine, National University of Singapore, 16 Medical Drive, Singapore, 117600 Singapore

**Keywords:** Chronic pain, Osteoarthritis, Interleukin-6, Cytokine

## Abstract

**Background:**

Chronic osteoarthritic pain is not well understood in terms of its pathophysiological mechanism. Activated glial cells are thought to play a role in the maintenance of chronic pain. T98G glioblastoma cell line was previously observed to release higher amounts of interleukin-6 (IL-6) when treated with cerebrospinal fluid (CSF) from patients with another chronic pain condition, post-herpetic neuralgia. In this study, we investigated the ability of CSF from patients diagnosed with knee osteoarthritis suffering from chronic pain, to trigger the release of pro-inflammatory cytokines, IL-6, IL-1beta and tumour necrosis factor alpha (TNF-α) from T98G. Characterization of upstream signalling was also explored.

**Methods:**

Fifteen osteoarthritis patients undergoing total knee replacement due to chronic knee pain and 15 patients without pain undergoing other surgeries with spinal anaesthesia were prospectively recruited. CSF was collected during anaesthesia. CSF were added to cultured T98G cells in the presence of lipopolysaccharide. IL-6, IL-1β and TNF-α release from T98G cells were measured using enzyme immunoassay. Antibody array and western blotting were performed using CSF-triggered T98G cell lysates to identify possible signalling targets. Age, gender and pain scores were recorded. Mann-Whitney U test was used to compare IL-6 release and protein expression between groups. Association between IL-6 and pain score was analysed using linear regression.

**Results:**

Significant higher levels of IL-6 were released by T98G cells when induced by osteoarthritis patients’ CSF in the presence of LPS. The IL-6 levels showed positive association with pain score (adjusted B estimate = 10.1 (95% Confidence Interval 4.3–15.9); *p =* 0.001). Antibody array conducted with 6 pooled T98G cell lysate induced with osteoarthritis pain patient CSF identified greater than 2-fold proteins including STE20-related kinase adaptor protein and spleen tyrosine kinase. Further validation done using western blotting of individual CSF-triggered T98G cell lysate showed non-significant increase.

**Conclusion:**

Higher IL-6 release from T98G when triggered by OA-CSF, in the presence of LPS, suggest the presence of “unknown molecule” in CSF that may be crucial in the maintenance phase of chronic pain in our osteoarthritis population. Further studies on the signalling pathways involved in pain and relevance of IL-6 release from T98G cells in other pain models are needed.

## Background

Osteoarthritis (OA) is a common joint degenerative disease affecting many patients worldwide. It is characterised by the progressive loss of articular cartilage, joint space narrowing, secondary bone hyperplasia and atrophy of joint muscles [1]. This disease affects weight-bearing joints and is more severe in the hips and knees. The hallmark symptom in OA frequently experienced by patients is localised chronic pain, which reduces their quality of life [2]. There is currently no cure for knee OA and the available treatments include use of medications, physical therapy or surgery that involves total joint replacement [3, 4]. Despite having medications to alleviate pain, chronic pain is still a major problem for OA patients.

There is a lack of understanding about the signalling pathways involved in pain. Mechanisms underlying chronic pain may involve activation of a series of signalling molecules leading to increased peripheral and/or central sensitisation. Some studies evaluated inflammatory cytokines such as tumour necrosis factor alpha (TNFα), interleukin-1 beta (IL-1β), IL-6 and IL-8 and IL-10 in patients with chronic pain-related diseases. For instance, IL-6 is elevated in cerebrospinal fluid (CSF) of patients with complex regional pain syndrome (CRPS) [5] and elevated IL-8 in patients with fibromyalgia [6].

Other studies showed chronic pain in OA being mediated by changes in neurotrophic factors, chemokines and interleukins as a response by chondrocytes or synovial cells at the joint in the synovial fluid [2]. Some showed glia activation related to pathogenesis of chronic pain [7]. Besides supporting neuronal cells, glial cells are found to have key roles in pain modulation. The exact role has not been elucidated [8].

Our group previously showed that CSF of post-herpetic neuralgia (PHN) chronic pain patients induced elevated IL-6 release from human glioblastoma T98G cells [9]. Therefore, we hypothesised that IL-6 is crucial in the maintenance phase of chronic pain signalling. In this study, we aimed to investigate whether CSF from a different chronic pain model (i.e. knee OA) could also induce the release of pro-inflammatory cytokines IL-6, IL-1β and TNFα from T98G cells. In addition, we also investigate possible signalling pathways involved in chronic OA pain.

## Methods

### Study design and patient population

The procedure for CSF collection was approved by our Institutional Ethics Board and written informed consent was obtained from each patient (DSRB Ref No. 2011/01710). Patients (both the pain group and the group without pain) were prospectively recruited by the attending anaesthesiologists prior to surgery. All patients recruited in the pain group satisfied the following inclusion criteria: (a) Confirmed OA of knees from history and x-ray of the knees by orthopaedic surgeons, (b) Numeric pain rating scale of more than 3 (0 being no pain; 10 being worst possible imaginable pain), (c) Knee pain of more than 6 months duration, (d) Scheduled for total knee replacement surgery under spinal anaesthesia, (e) Age of 21 years and above, (f) No other concurrent chronic pain conditions. Patients without pain (NP) and undergoing surgery with spinal anaesthesia served as the control group. The inclusion criteria for no pain (NP) group include: (a) Patients who do not have knee osteoarthritis, (b) No history of any type of chronic pain conditions over the past 1 year; (c) Patient consented for surgery below the umbilicus under a spinal anaesthesia, (d) Age of 21 years and above. Pregnant patients or patients with severe end-organ function impairment (e.g. heart, lungs, liver and kidneys) were excluded.

CSF was obtained during the conduct of spinal anaesthesia, where two-ml of CSF was withdrawn after the dura was punctured, before injecting local anaesthetic into the subarachnoid space to effect the spinal anaesthesia. A total of 15 chronic knee OA patients and 15 NP patients were recruited. CSF samples were then stored at − 80 °C. Patients’ demographics such as gender and age were also recorded.

### Measurement of pro-inflammatory cytokine release in cell culture

Human glioblastoma cell line T98G (ATCC-CRL1690, American Type Culture Collection, Manassas, VA) was cultured as previously described [9]. In brief, Eagle’s Minimum Essential Medium (Gibco, Grand Island, New York) (EMEM) containing 10% fetal bovine serum (FBS) (HyClone, Utah, USA) and 1X antibiotic-anti-mycotic cocktail (HyClone, Utah, USA) was used.

T98G cells were grown to approximately 90% confluency in 12-well plates. CSF from patients were filtered using a 0.22 μm filter to remove any existing cells in the CSF prior to all subsequent experiments. Filtered CSF was added into cultured T98G cells. The culture medium was replaced with FBS-free EMEM medium before addition of 50 μl CSF along with 32 μg/ml lipopolysaccharide (LPS) from *Escherichia coli* 055:B5 (Sigma Aldrich, St Louis MO, USA) to the T98G cells. Cells were induced for 48 h at 37 °C and 5% CO_2_ environment. LPS was used to enhance cytokine release from the cells [9, 10]. Each well with the trigger of 50 μl CSF was from individual patient. After 48 h, the medium containing “released cytokines” was collected and the amount of IL-6 in this supernatant was measured in duplicates using enzyme-linked immunosorbent assay (ELISA) according to manufacturer’s instructions (DY206, R&D Systems, Minneapolis, USA). The detection range for the IL-6 standard used was between 9.38 pg/ml to 600 pg/ml.

Using supernatant from the above 48 h CSF-triggered T98G protocol, TNFα and IL-1β levels were also determined with ELISA assays- Human TNFα Duoset ELISA (DY210) and Human IL-1β/IL-1F2 Duoset (DY201) respectively (R&D Systems, Minneapolis, USA). Detection range of standards for TNFα was 15.6 pg/ml to 1000 pg/ml; and IL-1β was 3.91 pg/ml to 250 pg/ml.

### Antibody array

Nuclear factor kappa light chain enhancer of activated B cells (NF-κB) phospho antibody array (PNK215, Fullmoon Biosystems, CA, USA) was used to screen for changes in protein expression and phosphorylation profile in our samples. This array applied an ELISA-based technique where samples were biotinylated before adding to the array slide containing the affixed antibodies. Biotin on samples upon interaction with dye-labelled streptavidin on array would generate the fluorescence signal. Patient CSF-triggered T98G cells, in the presence of LPS, were harvested and lysed with CelLytic MT Cell Lysis Reagent (C3228; Sigma Aldrich). Total protein from these T98G cell lysate were quantified using bicinchoninic acid (BCA) assay (Pierce BCA Protein Assay Kit, Thermo Scientific, USA). Due to limited amount of CSF drawn from each patient, and to obtain a minimum amount of protein for each antibody array, the triggered T98G cell lysates from OA pain samples (*n* = 6) were pooled in equal quantity and added to the antibody array slide. Similarly, the same amount of T98G cell lysates from NP samples (n = 6) were pooled and added to another antibody array slide. The assay was then performed according to manufacturer’s instructions. Array image was captured using array scanner (GenePix 4000B; Molecular Probes, CA, USA). Analysis of array data was done using Genescan software. Comparison of signals between pooled OA pain sample and pooled NP sample was done after normalization with glyceraldehyde-3-phosphate dehydrogenase (GAPDH).

### Western blot analysis

To validate the fold increase protein expression and phosphorylation changes as observed in the antibody array for OA triggered T98G cells, all individual OA and NP-CSF triggered T98G cell lysates (*n* = 15 each) were checked for protein expression level of targets with western blotting. Ten micrograms of each CSF-triggered T98G cell lysates were loaded per lane of 10% Tris-glycine gels and run using Sodium dodecyl sulphate polyacrylamide gel electrophoresis (SDS-PAGE). Proteins on gel were transferred onto Polyvinylidene difluoride (PVDF) membrane. 0.1% Tween-20 added into Tris-buffered saline (TBS-T) was used to wash membranes and prepare antibodies. Non-specific binding sites on the membranes were blocked in 5% w/v Bovine serum albumin (BSA)-TBS-T or milk-TBS-T. BSA was used when checking for spleen tyrosine kinase (Syk); while milk was used for STE20-related kinase adaptor protein (STRAD) and GAPDH. Primary antibodies used in this study are: STRAD (G-8) (Santa Cruz Biotechnology, CA, USA); Syk (D3Z1E) (Cell Signaling Technology Inc., MA, USA); and GAPDH (MAB374) (EMD Millipore, Darmstadt, Germany). GAPDH, with a molecular weight of 37 kDa, was used as the loading control for normalization. Membranes were imaged with ChemiDoc XRS+ system. Densitometry quantification was done with ImageLab software.

### Statistical analysis

All data were analysed using GraphPad Prism v5.0 (GraphPad, La Jolla, CA, USA) and SPSS v24.0 (IBM SPSS, Armonk NY, USA). Cytokine levels was checked for normality and deemed not normally distributed, thus non-parametric Mann Whitney-U test was used. Mann-Whitney U test was also used to analyse STRAD and Syk protein levels from western blotting between groups. Simple and multivariate linear regression model analyses were used to study the relationship of pain score and cytokine level. A *p*-value of less than 0.05 was considered statistically significant.

## Results

### Patient demographics

The average age of patients in our cohort is 61 ± 13 years. Demographics of OA pain and NP patients are described in Table 1. The control group are patients without pain. The types of surgical procedures for the NP group included transurethral resection of the prostate (*n* = 7), hernia repair (*n* = 2) and ligation of piles (*n* = 6).

**Table 1:** Patient demographics of study groups

Group	OA pain (*n* = 15)	No pain (*n* = 15)	*p*-value
Age (years)	60 ± 13	63 ± 14	0.618
Gender	7 male, 8 female	14 male, 1 female	0.014
Pain Score	7 ± 2	0	< 0.001
History of diabetes mellitus, yes	3 (20%)	5 (33.3%)	0.682
History of hypertension, yes	8 (53.3%)	8 (53.3%)	1
History of peripheral vascular disease, yes	0	0	–
History of hyperlipidaemia, yes	6 (40%)	8 (53.3%)	0.715

Data are presented as Mean ± standard deviation (SD) or number (percentage). *p*-value of 0.05 is considered statistically significant

### IL-6 release from T98G cells

The IL-6 levels in the CSF samples from OA and NP patients were below the lowest detection limit of the ELISA kit (9.38 pg/ml) used. In order to detect IL-6 levels using the ELISA method, the additional induction methodology used in [9] were used to amplify the IL-6 changes. Briefly, CSF was used to induce T98G cells in the presence of LPS in order for the T98G cells to release IL-6 at levels measurable using the ELISA method.

**Fig. 1:**
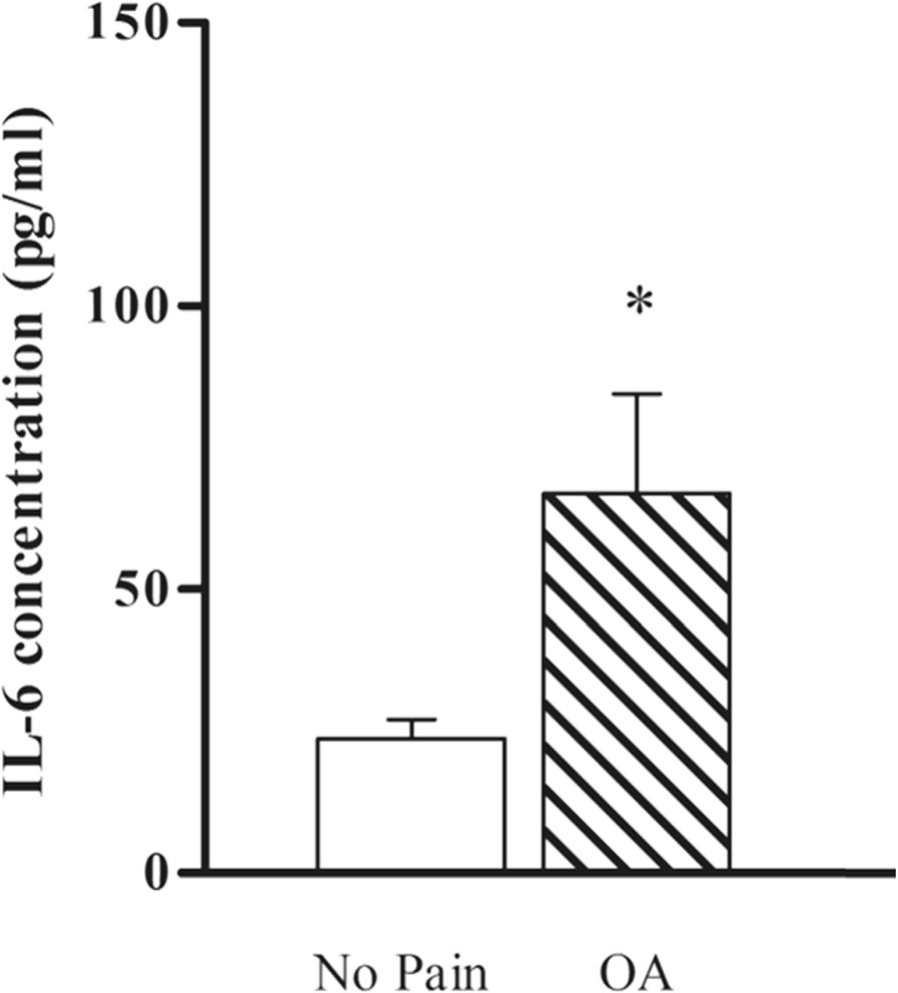
Higher IL-6 release from OA pain CSF stimulated T98G cells, in the presence of LPS. Concentration of IL-6 release from T98G cells when triggered with 50 μl CSF of either OA-CSF or NP-CSF, in the presence of LPS. Bars are plotted as mean ± standard error of mean (15 patients per group), *p < 0.05 when using Mann Whitney-U test

In the absence of CSF, addition of LPS to T98G cells did not trigger detectable levels of IL-6 release [9]. When both patients’ CSF and LPS were added to T98G cells, IL-6 release was detected in the cell culture media. 67.0 ± 17.7 pg/ml of IL-6 was detected when induced with OA-CSF (*n* = 15) whereas 23.7 ± 3.4 pg/ml of IL-6 was detected for NP-CSF group (n = 15). There was a statistically significant higher amount of IL-6 released from T98G cells triggered with OA-CSF as compared to NP-CSF (Fig. 1; Mann Whitney-U test, *p* = 0.002).

### TNFα and IL-1β release from T98G cells

TNFα and IL-1β (lowest detection range of 15.6 pg/ml and 3.91 pg/ml respectively), were evaluated and found to be undetectable in T98G cell culture media when induced with CSF (OA/NP) and LPS, with their respective ELISA kits.

### Relationship between IL-6 release and pain score

There was a significant positive relationship between pain score and IL-6 cytokine levels (unadjusted B estimate = 7.1 (95% Confidence Interval (C.I.) = 2.1–12.1); *p* = 0.007). After adjusting for age and gender in the multivariate model, the significant association persists (adjusted B estimate = 10.1 (95% CI = 4.3–15.9); *p* = 0.001).

### Possible targets found in antibody array

**Fig. 2:**
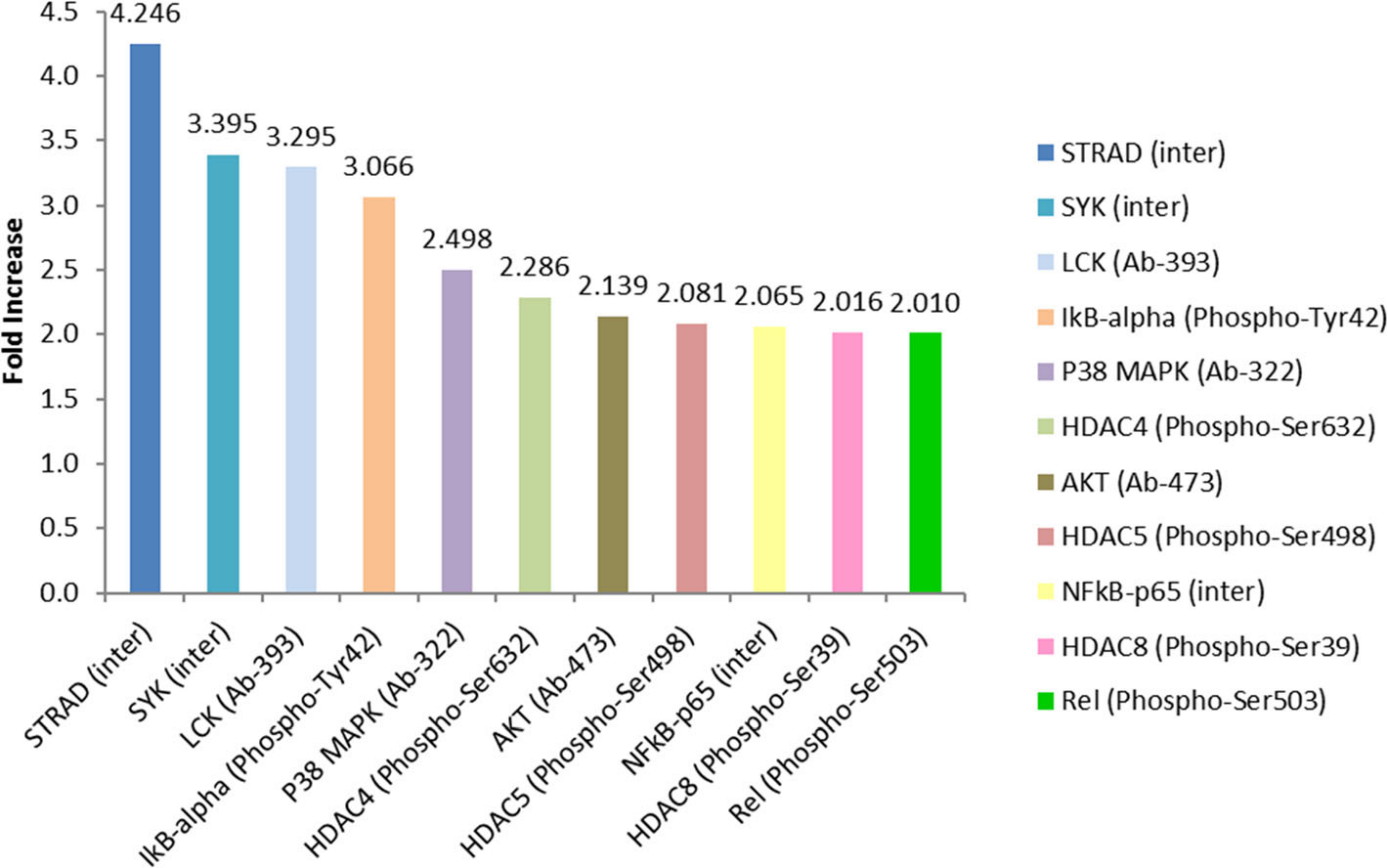
Protein expression and phosphorylation changes in NF-κB related signalling pathways assayed using an antibody array. Pooled protein lysates from OA-CSF triggered T98G cell (*n* = 6) with the highest pain score and NP-CSF triggered T98G cell (*n* = 6) were assayed using the array. Proteins with expression or phosphorylation increase for more than 2-fold in OA-CSF triggered T98G lysate were plotted on the graph

The antibody array used consisted of 6 replicates of 215 antibodies for targets related to the NF-κB pathway. Forty microgram of T98G cell lysates, harvested from each of the 6 individual CSF samples trigger, in the presence of LPS, were pooled together to give a total 240 μg protein for the array testing. The 240 μg pooled OA-CSF triggered T98G cell lysate was added to one array slide; while pooled NP-CSF triggered T98G cell lysate was added to another slide. The fluorescence signal of all 6 antibody replicates within each array were recorded and the average was calculated. GAPDH was used to normalise the signal for both OA and NP samples. Those with a greater than 2-fold increase in OA-CSF triggered T98G cell lysate as compared to that of NP-CSF were noted. The more notable ones having greater than 3-fold increase were STRAD (4.25 fold), Syk (3.40 fold), lymphocyte-specific protein tyrosine kinase (3.30 fold) and IkappaB-alpha (3.07 fold) (Fig. 2). STRAD and Syk were chosen for further validation using western blotting.

### Protein expression of STRAD and Syk

**Fig. 3:**
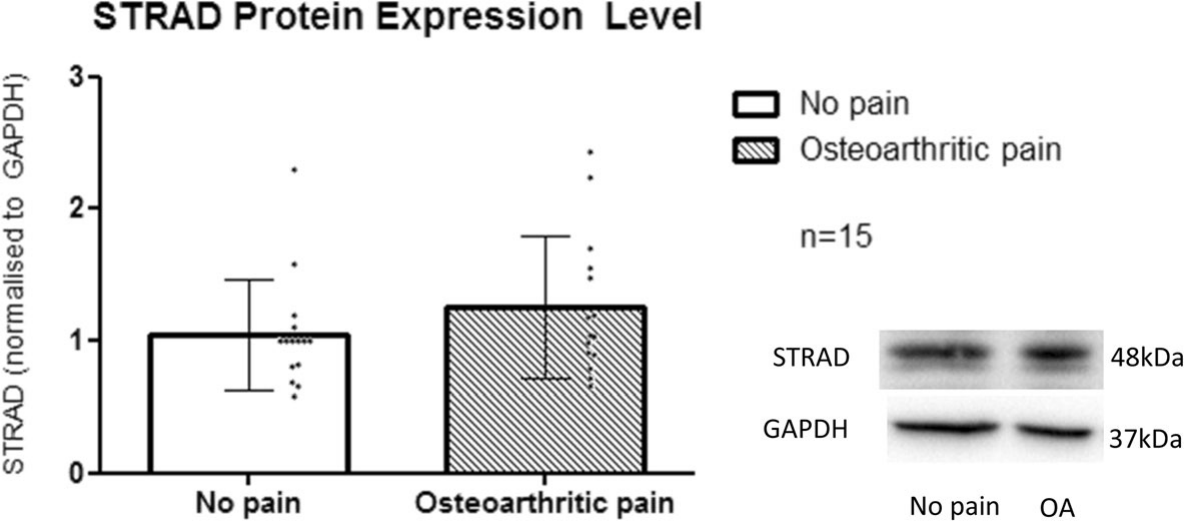
Normalized STRAD protein expression level between the study groups. Western blotting was run and each dot represents STRAD protein expression of each sample of individual patient’s CSF induction in T98G cells. Bars are plotted as mean ± SD (15 patients per group), *p < 0.05 when using Mann Whitney-U test

The molecular weight of STRAD is 48 kDa. For STRAD protein, there is a higher protein expression level in OA patients (1.26 ± 0.54; *n* = 15) as compared to NP patients (1.05 ± 0.42; n = 15). However, the difference was not statistically significant (*p* = 0.298) (Fig. 3).

**Fig. 4:**
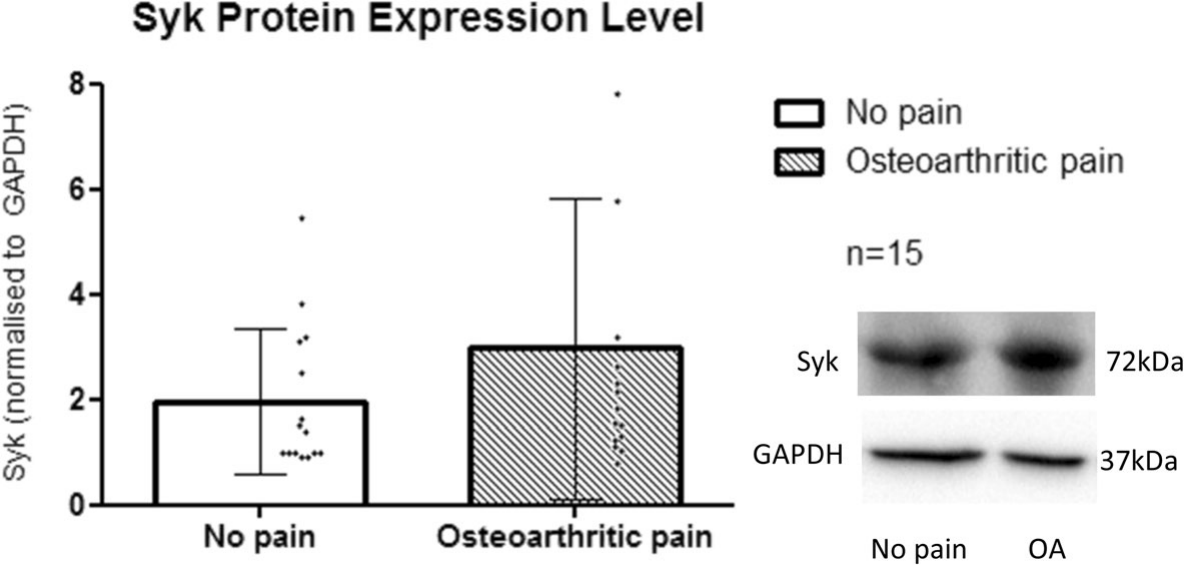
Normalized Syk protein expression level between the study groups. Western blotting was run and each dot represents Syk protein expression of each sample of individual patient’s CSF induction in T98G cells. Bars are plotted as mean ± SD (15 patients per group), *p < 0.05 when using Mann Whitney-U test

Syk has a molecular weight of 72 kDa. Similarly, higher protein expression level was observed in OA patients (2.98 ± 2.9; n = 15) as compared to NP patients (1.96 ± 1.4; n = 15) for Syk protein. This higher protein expression in OA patients was not statistically significant (*p* = 0.183) (Fig. 4).

## Discussion

IL-6 is a multi-functional protein cytokine known to play a role in the modulation of signalling in the central nervous system (CNS) such as those influencing immune responses, inflammation and wounds [11]. IL-6 has been implicated in various diseases related to pathological pain. It is generally believed that IL-6 release by the activated glial cells particularly the microglia and the astrocytes following painful stimuli [12]. Here, we showed that CSF obtained from a cohort of patients with chronic knee pain secondary to knee osteoarthritis when added together with LPS, was able to stimulate T98G cells to release IL-6. Whilst under similar condition, CSF obtained from a cohort of NP patients provoke significantly lesser IL-6 release. LPS alone did not stimulate T98G cells to release IL-6, and IL-6 was not detected in any CSF sample prior to the cell culture experiments. This is also supported by the positive correlation between patient’s numeric pain scores and the amount of IL-6 released. Taken together, our results suggest that CSF obtained from patients suffering from chronic OA knee pain, contain an as-yet-unknown molecule(s) which can trigger IL-6 release from cultured T98G cells. This result is also consistent with our previous published work using CSF obtained from another cohort of chronic pain patients (post-herpetic neuralgia).

We explored the signalling pathway upstream of IL-6 in our T98G model. Specifically, NF-κB explorer array was chosen as it is one of the common upstream molecular players of IL-6. Several protein targets were found to be elevated with greater than two-fold in OA-CSF triggered T98G cells as compared to NP-CSF triggered ones (Fig. 2), these targets could be important in delineating the chronic pain signalling mechanism.

Two protein targets which showed an increase of 4.2 and 3.4 fold in OA-CSF in the array were STRAD and Syk respectively. STRAD, predominantly found in CNS, is known to complex with serine/threonine kinase LKB1 and scaffold protein MO25α to activate AMP-activated protein kinase (AMPK) signalling pathway which includes release of pro-inflammatory cytokines [13]. Syk plays a role in signal transduction influencing cells of the immune system- both B and T cells [14]. NF-κB and IL-6 are downstream of AMPK [15]. Although both STRAD and Syk showed the highest expression among other potential target proteins in the pooled T98G cell lysate following exposure to CSF from 6 OA patients with the highest pain score, when all samples were further validated individually in western blotting, their differences were not statistically significant when compared with samples from the NP group (Figs. 3 and 4). Several possibilities to explain our result are: (a) small sample size, (b) wider range of patients’ visual analog scale (VAS) pain scores in the 15 OA samples when tested in western blotting versus the pooled 6 OA samples with the highest VAS pain scores used on the array analysis.

The strength of our study is the use of human CSF to provide an objective evaluation of chronic pain. CSF, as compared to peripheral blood, has a more direct contact with the CNS environment. It bathes the CNS and therefore the contents in CSF may be crucial in understanding changes of the underlying disease associated with chronic knee pain [16]. Our findings may lead to better understanding of the pathogenesis of OA knee pain and future therapeutic approaches if the identity of the pain signalling molecule and the upstream signalling pathway(s) can be elucidated.

Although we found that a 48-h treatment time point for measuring IL-6 release provides sufficient signal strength in the ELISA, it may not be the peak protein expression or phosphorylation changes for STRAD and Syk, thus leading to overall lack of significance in western blotting results. Another limitation was that only a limited number of antibodies were tested.

## Conclusion

In summary, CSF from chronic knee pain patients suffering from OA knees was able to induce higher levels of IL-6 release from T98G cells than NP-CSF in the presence of LPS. This finding suggests the presence of an as-yet-unknown molecule(s) in the CSF of patients with chronic pain which could potentially serve as clinically informative biomarker(s). Further studies are needed to investigate the relevance of IL-6 release by T98G glial cells in other chronic pain models and to evaluate any potential upstream players.

## Data Availability

The datasets generated and analysed during the current study are available from the corresponding author on reasonable request.

## Abbreviations

AMPK: AMP-activated protein kinase
ATCC: American Type Culture Collection
BCA: Bicinchoninic acid
BSA: Bovine serum albumin
CI: Confidence Interval
CNS: Central Nervous System
CRPS: Complex Regional Pain Syndrome
CSF: Cerebrospinal fluid
ELISA: Enzyme-linked immunosorbent assay
EMEM: Eagle’s minimum essential medium
F: Female
FBS: Fetal Bovine Serum
GAPDH: Glyceraldehyde-3-phosphate dehydrogenase
IL: Interleukin
LPS: Lipopolysaccharide
M: Male
NF-κB: Nuclear factor kappa light chain enhancer of activated B cells
NP: No pain
OA: Osteoarthritis
PHN: Post-herpetic Neuralgia
SD: Standard deviation
SDS-PAGE: Sodium dodecyl sulphate polyacrylamide gel electrophoresis
STRAD: STE20-related kinase adaptor protein
Syk: Spleen tyrosine kinase
TNFα: Tumour Necrosis Factor-alpha
VAS: Visual Analog Scale
